# Supplementary figures and images for: A Second Role for the Second Messenger Cyclic-di-GMP in E. coli: Arresting Cell Growth by Altering Metabolic Flow
Source: mBio. 2023 Apr 10;14(2):e00619-23. doi: 10.1128/mbio.00619-23 (PMC10127611; doi:10.1128/mbio.00619-23)

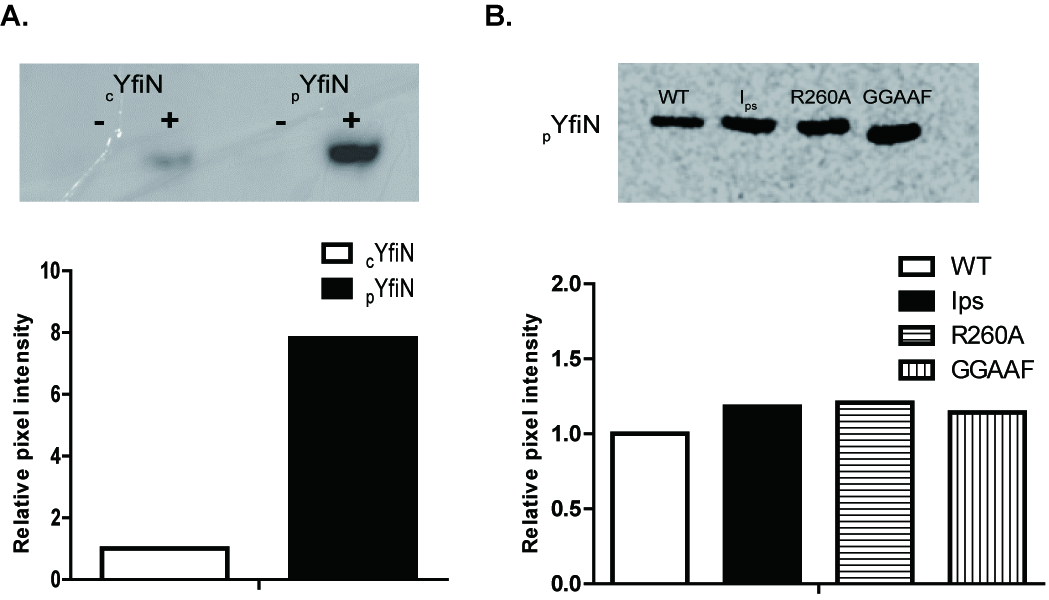

Supplement: FIG S1 [file mbio.00619-23-s0002.tif]

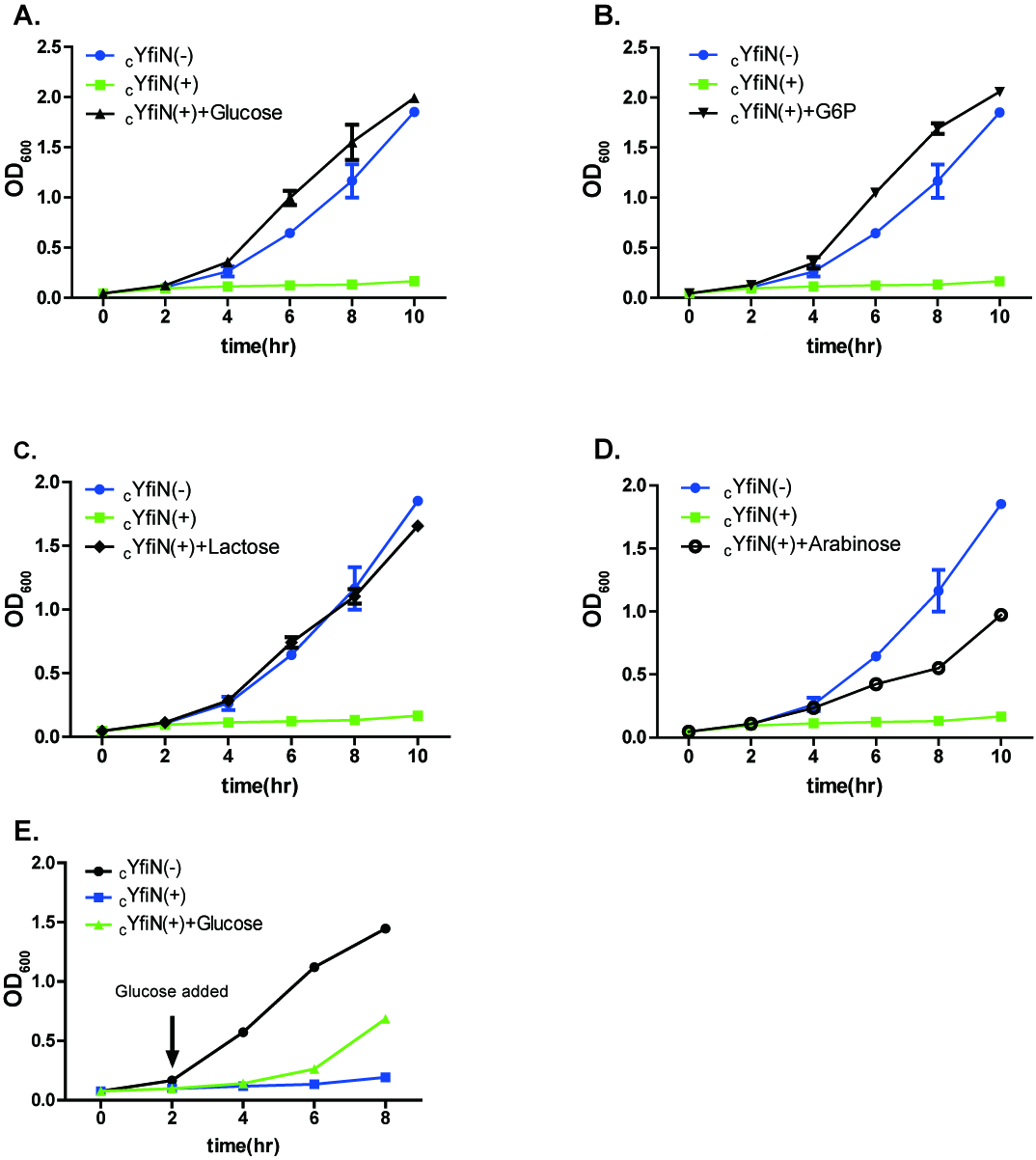

Supplement: FIG S2 [file mbio.00619-23-s0003.tif]

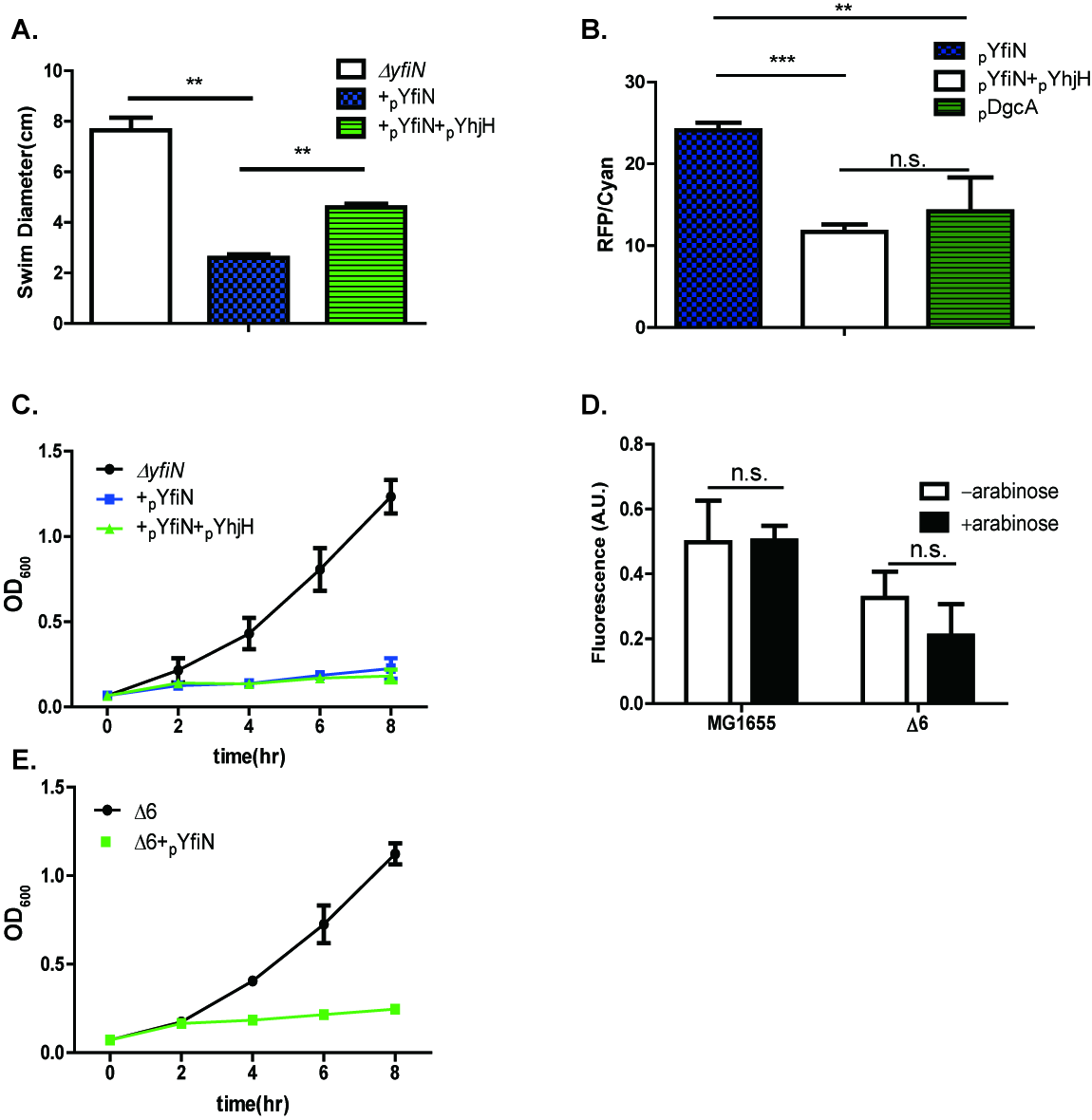

Supplement: FIG S3 [file mbio.00619-23-s0004.tif]

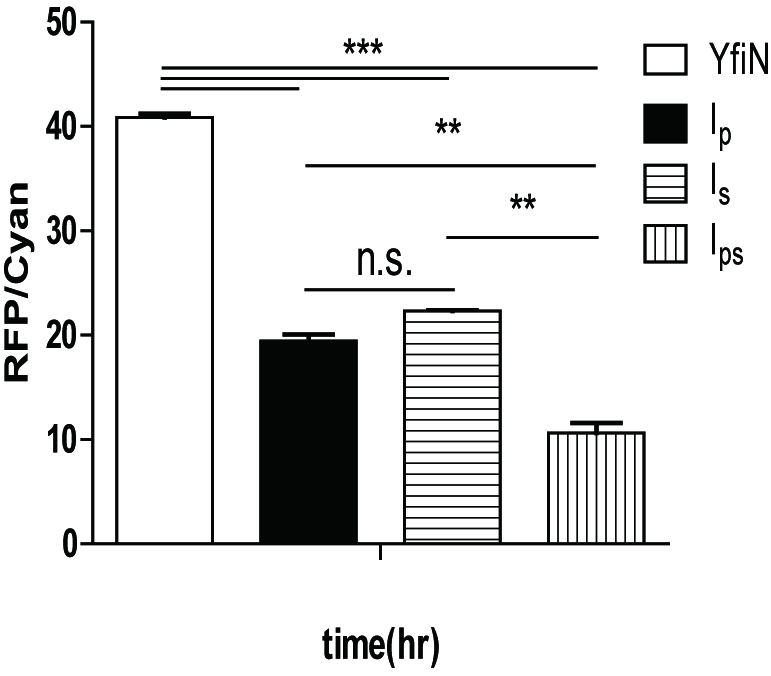

Supplement: FIG S4 [file mbio.00619-23-s0005.tif]

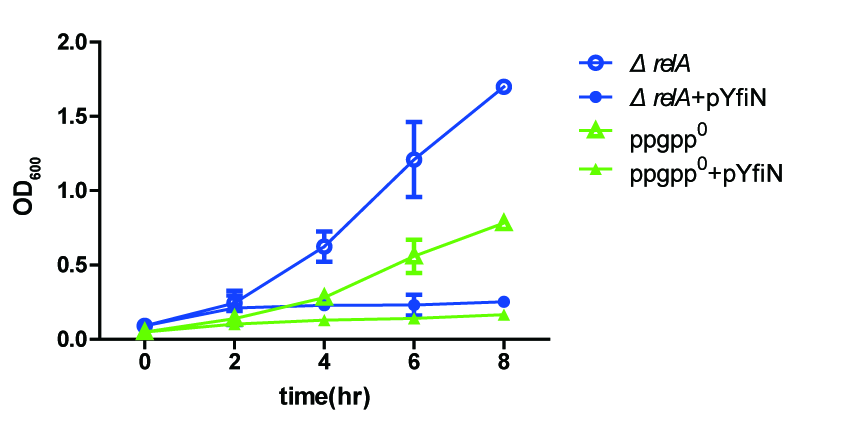

Supplement: FIG S5 [file mbio.00619-23-s0006.tif]

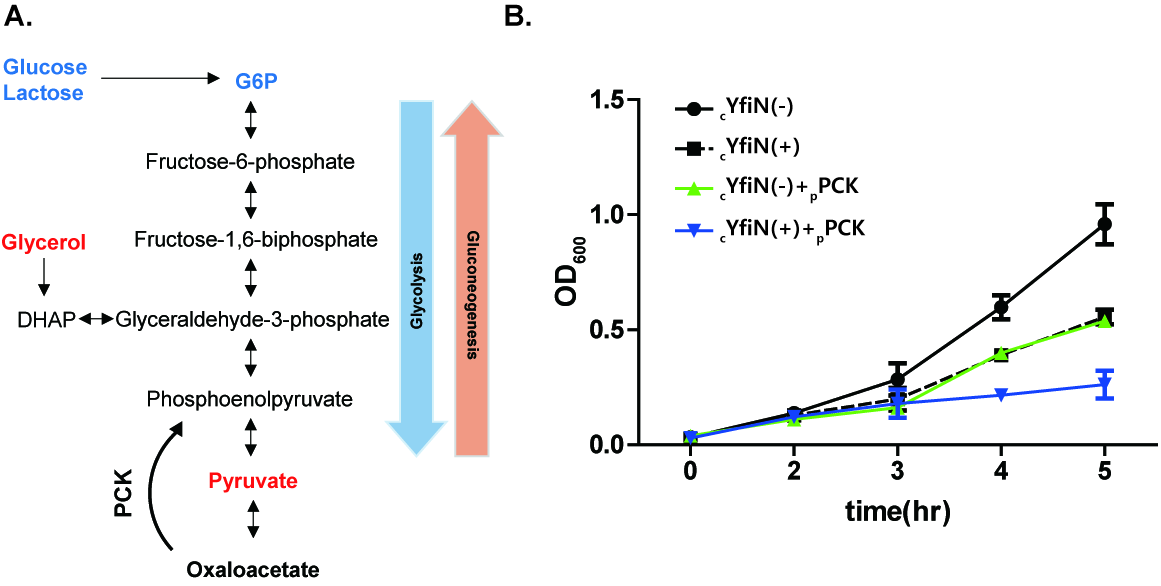

Supplement: FIG S6 [file mbio.00619-23-s0007.tif]

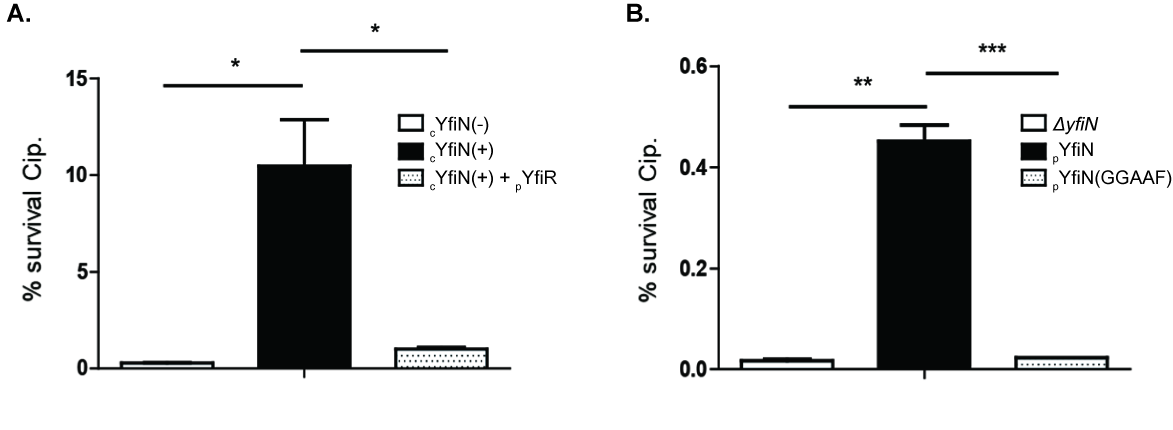

Supplement: FIG S7 [file mbio.00619-23-s0008.tif]

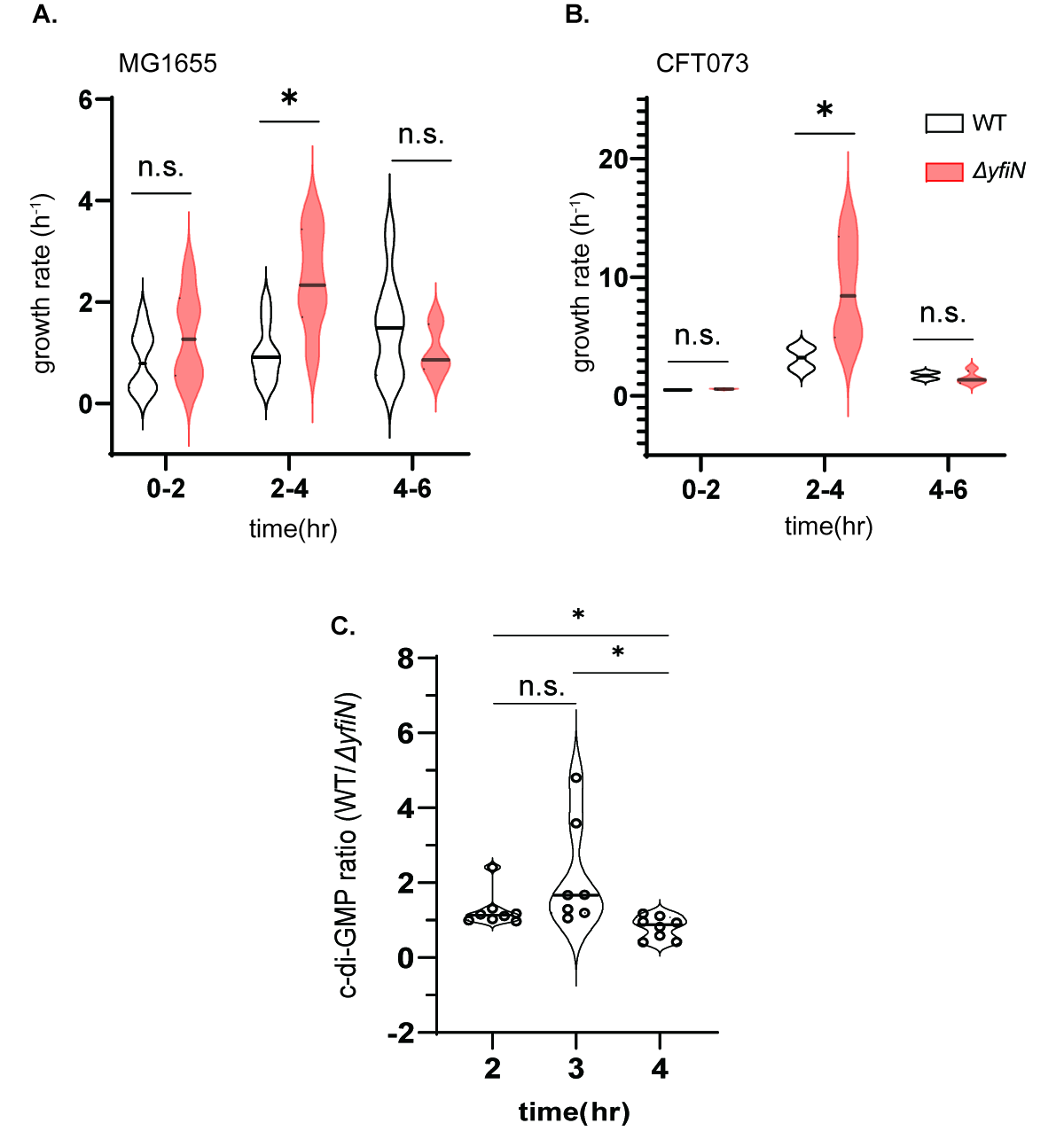

Supplement: FIG S8 [file mbio.00619-23-s0009.tif]

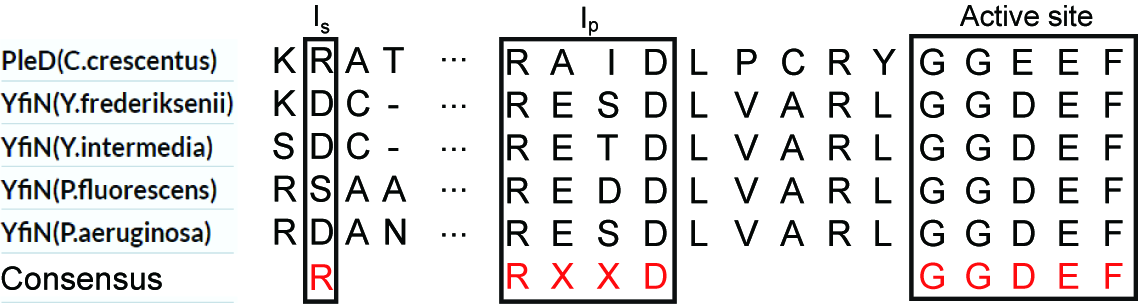

Supplement: FIG S9 [file mbio.00619-23-s0010.tif]
