# Supplementary material for: A Second Role for the Second Messenger Cyclic-di-GMP in E. coli: Arresting Cell Growth by Altering Metabolic Flow
Source: mBio. 2023 Apr 10;14(2):e00619-23. doi: 10.1128/mbio.00619-23 (PMC10127611; doi:10.1128/mbio.00619-23)
Supplement: TABLE S1 [file mbio.00619-23-s0001.docx]

**Table S1**

| **Strains** | **Genotype or Description** | **Reference** |
| --- | --- | --- |
| MG1655 | K12 wild-type (WT) strain: F^-^ , λ^-^ , rph-1 | Laboratory Collection |
| HK359 | MG1655 ∆*yfiN* | [24] |
| HK461 | HK359 + pBAD30_YfiN_GFP_ | [24] |
| JH101 | HK359 + pBAD30_YfiN | This study |
| HK532 | MG1655 yfiR::kan(🡨) yfiN::pBAD-*yfiN*-*gfp* | [24] |
| HK533 | MG1655 yfiR::kan(🡨) yfiN::pTrc-*yfiN*-*gfp* | This study |
| HK800 | HK359 + pBAD33_YfiN_GFP_ |  |
| JH102 | MG1655 + pBAD33_DgcA | This study |
| JH100 | HK359 + pBAD30_YfiN | This study |
| JH103 | HK359 + pBAD30_YfiN(GGAAF) | This study |
| JH104 | HK359 + pBAD30_YfiN(I_p_) | This study |
| JH105 | HK359 + pBAD30_YfiN(I_s_) | This study |
| JH106 | HK359 + pBAD30_YfiN(I_ps_) | This study |
| JH107 | MG1655 + pBAD33_DgcA(GESD) | This study |
| JH108 | HK359 + pBAD33_YfiN(R260A)_GFP_ | This study |
| JH109 | HK532 + ASKA_PCK | This study |
| JH202 | MG1655 + pBAD33_DgcC | This study |
| JH203 | MG1655 + pBAD33_DgcE | This study |
| JH204 | MG1655 + pBAD33_DgcF | This study |
| JH201 | MG1655 + pBAD33_DgcJ | This study |
| JP1932 | AW405 Δ*bcsA csgD pgaC fimA wcaD yjbE* | [32] |
| JH110 | HK359 + pBAD33_YfiN + pTrc99a_YhjH | This study |
| JH111 | HK532 + pTrc99a_YfiR | This study |
| JH250 | HK359 + pBAD30_YfiN-Flag (c-term.) | This study |
| JH251 | HK359 + pBAD30_YfiN(I_ps_)-Flag (c-term.) | This study |
| JH252 | HK359 + pBAD30_YfiN(GGAAF)-Flag (c-term.) | This study |
| JH253 | HK359 + pBAD30_YfiN(R260A)-Flag (c-term.) | This study |
| JH300 | HK359 + pBAD30_YfiN-Flag(c-term.) | This study |
| JH301 | HK359 + pBAD30_YfiN + pFY4535 | This study |
| JH302 | HK359 + pBAD30_YfiN(I_p_) + pFY4535 | This study |
| JH303 | HK359 + pBAD30_YfiN(I_s_) + pFY4535 | This study |
| JH304 | HK359 + pBAD30_YfiN(I_ps_) + pFY4535 | This study |
| JH305 | MG1655 + pFY4535 | This study |
| JH306 | HK359 + pFY4535 | This study |
| JH307 | JH110 + pFY4535 | This study |
| JH308 | HK359 + pBAD33_YfiN + pTrc99a_Empty + pFY4535 | This study |
| JH309 | JH102 + pFY4535 | This study |
| CFT073 | Uropathogenic strain of *E. coli* | R. Welch, U. Wisconsin-Madison |
| HK701 | CFT073∆*yfiN* | Hyo Kyung Kim |
|  |  |  |
| Plasmids | Expressed Proteins | Reference |
| pKD4 | Kanamycin resistance gene template | [66] |
| pKD46 | λ Red Recombinase | [66] |
| pCP20 | FLP recombinase | [66, 69] |
| pTrc99a | Cloning vector, P_Trc_; Amp^R^ | [69, 70] |
| pBAD30 | Cloning vector; P_BAD_; Amp^R^ | [70] |
| pBAD33 | Cloning vector; P_BAD_; Cm^R^ | [24, 70] |
| pBAD30_YfiN_GFP_ | pBAD::YfiN-GFP(C-terminal) | [24] |
| pBAD30_YfiN | pBAD::YfiN | This study |
| pBAD33_YfiN | pBAD:;YfiN | This study |
| pBAD30_YfiN(GGAAF) | pBAD:: YfiN (D329A E330A) | This study |
| pBAD30_YfiN(Ip) | pBAD::YfiN (G317R H320D) | This study |
| pBAD30_YfiN(Is) | pBAD::YfiN (N280R) | This study |
| pBAD30_YfiN(Ips) | pBAD::YfiN (N280R G317R H320D) | This study |
| pBAD33_DgcA | pBAD::DgcA | This study |
| pBAD33_DgcA(GESD) | pBAD::DgcA (R80G) | This study |
| pBAD30_YfiN(R260A)_GFP_ | pBAD::YfiN (R260A)-GFP | Hyo Kyung Kim |
| pBAD30_YfiN-Flag (c-term.) | pBAD::YfiN-Flag | This study |
| pBAD30_YfiN(I_ps_)-Flag (c-term.) | pBAD:: YfiN(I_ps_)-Flag | This study |
| pBAD30_YfiN(GGAAF)-Flag (c-term.) | pBAD:: YfiN(GGAAF)-Flag | This study |
| pBAD30_YfiN(R260A)-Flag (c-term.) | pBAD:: YfiN(R260A)-Flag | This study |
| pFY4535_pmmb-bc3-5-phok-sok | pMMBGent::c-di-GMP sensor | [34] |
| pBAD33_DgcC | pBAD::DgcC | This study |
| pBAD33_DgcE | pBAD::DgcE | This study |
| pBAD33_DgcF | pBAD::DgcF | This study |
| pBAD33_DgcJ | pBAD::DgcJ | This study |
| pTrc99a_Empty | pTrc99a::Empty | This study |
| pTrc99a_YhjH | pTrc::YhjH | This study |
| pTrc99a_YfiR | pTrc::YfiR | This study |
